# Chemistry and Medicine in Close Cooperation; or a View of the Latest Progress of Organic Chemistry in Relation to Experimental and Speculative Medical Research, Intended as a Complete Text-Book for the Study of Organic Chemistry Generally, and Particularly in the Domain of Medicine and Pharmacy, as Also Materia Medica

**Published:** 1843-07

**Authors:** 


### ART. VIII.

*Chemie und Medicin in ihrem engeren Zusammenwirken, oder Beduetung der neuren Fortschritte der organischen Chemie für erfahrungsmässige und speculative ärztliche Forschung, als vollständige Lehrschrift für die studien der organischen Chemie uberhaupt, insbesondere aber für die im Gebiete der Medicin und Pharmacie, so wie für die Fortschritte der Heilmittellehre.* Von Dr. F. L. HUENEFELD. In Zwei Büchern. —*Berlin*, 1841. 8vo, pp. 396, 372.

*Chemistry and Medicine in close cooperation; or a view of the latest progress of Organic Chemistry in relation to experimental and speculative medical research, intended as a complete text-book for the study of organic chemistry generally, and particularly in the domain of Medicine and Pharmacy, as also Materia Medica.* By Dr. F. L. HUENEFELD, Professor of Chemistry, Pharmacy, and Mineralogy at Gräfswald. In Two Books.—*Berlin*, 1841.

IT was justly remarked by the experienced Rudolphi, that "no mere chemist is competent to be the author of a treatise upon animal physiology."* Indeed we are thoroughly persuaded that those exact habits of research which qualify a man for investigating with success the changes of composition that occur among the constituent parts of different bodies, unfit him for grappling with a power so mysterious and inscrutable as that of life. Thus it is that the philosopher, in professing to account for vital processes on purely chemical principles, has tended rather to perplex than elucidate the subject. We are not prepared to deny that chemistry may materially aid, to a certain extent, in explaining various organic phenomena, but its sphere is limited. Were it otherwise, the laws of chemistry would be the laws of life.

It is no easy task to review the work before us, as it is extremely diffuse, and in some places contradictory. To have done ample justice to the wide field which the author embraces might have well occupied at least half a dozen such volumes. Hence the work is neither well suited for elementary study nor for reference. The first volume is little more than a *catalogue raisonnée* of vegetable and animal substances. It commences with a brief historical notice of the early iatro-chemical doctrines and of the action of remedies. The author then goes on to discuss the present state of organic chemistry; the *general* conditions and relations of the various substances, as their aggregate form, specific gravity, sensible properties, microscopic peculiarities, spontaneous resolution, modes of decay, and the like; next their *specific* relations as regards water, acids, alkalies, bases, metals, salts, and so on.

He ushers in his second volume with some remarks concerning the physico-chemical endowments of the organism, more especially elasticity and capillarity. In pointing out the composition of the arterial texture, allusion is made to the circumstance of the lining membrane being composed of the fibrinous protein, and hence not liable to be acted upon by weak alkaline solutions, since had it consisted of the albuminous protein, it could not have withstood for any length of time the incessant contact with alkaline blood. (p. 8.)

* Physiologie, Bd. i. p. 118.

The chemical apparatus of the animal body appears to be essentially the capillary system. Hence endosmose, which may be reckoned a modification of the capillary force, plays a conspicuous part. Animal membranes, it is observed, become more permeable for the passage of liquids, when freed from fat by means of ether. It is therefore hinted that the different amount of fat deposited in membranous textures may influence more or less the power of absorption. (p. 14.)

The chapter on assimilation contains some original researches upon the saliva, which are interesting so far as the subject has of late given rise to much controversy: 1. The saliva notably reddens delicate litmus paper in the early part of the day, and one or two hours before, but particularly after eating. The author employed thin post paper, which did not of itself redden litmus, imbued with a solution of litmus, previously treated with ether, then allowed to stand for a considerable time and afterwards filtered. 2. The saliva offers an alkaline reaction directly before and during the act of eating (the period of appetite and mastication). It is expedient that an adequate quantity of saliva be placed on the paper. In some individuals, particularly in the instance of robust labourers, the saliva was at other times almost always faintly alkaline. 3. The saliva of children and adults frequently renders delicate red litmus paper blue, chiefly in the forenoon and night. The last paper was prepared by suspending the other paper in an atmosphere impregnated with hydrochloric acid gas, until it acquired a faint but appreciable red tinge. 4. Saliva taken from the same person, while it strikes a red colour with the blue, also strikes a blue colour with the red litmus paper; except during the time of meals and of appetite, when the alkalescence invariably predominates. 5. The saliva of a child at the breast, six months old, did not affect either red or blue litmus. 6. Litmus paper, whether red or blue, to which saliva had been applied became after drying permanently red. (p. 44.)

The fixed redness of the litmus paper would seem to depend on the presence of some ammoniacal salt; either the lactate, the acetate, or the phosphate. But why the saliva should at certain times possess the property of rendering blue litmus red, and red litmus blue, is another question; it is undoubtedly a mixture of the buccal mucus and of the fluids of several glands—the parotid, the submaxillary, the sublingual; and these most likely afford secretions differing as regards acid and alkaline reaction If we consider therefore in a chemical and physiological point of view, that the normal saliva is what belongs to the process of mastication, or rather to the period of appetite, we shall find it to be alkaline, as can be distinctly proved by the blue colour imparted to the juice of bilberries, shortly before and during a repast. Our author thinks the reddish-blue powder of iris or violet petals, when put on the tongue or in the mouth, affords a still more delicate test. It is always reddened, unless at the above periods.

We are indebted to M. Mandl for some curious investigations into the chemical nature of the secretions in connexion with the nerves which go to the respective secernent organs. He says that the saliva has an alkaline reaction; and that all organs supplied with nerves from the cerebro-spinal system (in the present instance the *nervus trigeminis* and *facialis*) afford an alkaline secretion, while those furnished from the ganglionic system yield an acid secretion in the state of health. The alkaline condition of the saliva coincides with the formation of salivary concretions, and with the so-called tartareous crust upon the teeth. Mitscherlich detected chloride of calcium in the residuum of incinerated saliva.

The saliva, according to M. Donné, acquires an acid reaction in inflammatory states of the system and in cases of irregular menstruation, becoming again alkaline on the subsidence of the inflammation and the restoration of the catamenia.

"If we call to mind that Pelouze and Fremy have shown that mucous membranes have the power of transforming sugar of milk, dextrine, and the like, into lactic acid at the ordinary temperature of the human body, it appears very probable that this peculiar property may be exalted in cases of disease, more especially in those attended with inflammatory irritation; and that while the mucous membrane of the mouth pours out an acid which is more than neutralized, being rendered even alkaline by the proper saliva, still this alkalinity will be counteracted by the excessive formation of lactic acid. If we consider further, that certain vegetable salts are converted by animal membranes into carbonates of their respective bases, a thing which, according to Wöhler, takes place in the living organism, we see good reason for the employment of such medicines as tartrate of potash, citrate of potash, and the like, in the slighter forms of inflammatory irritation." (p. 49.)

Some pages are occupied with details of the examination of phthisical sputa, and the distinctive characters of pus-globules. As the blood-corpuscles, with their nuclei, dissolve and disappear in bile, so likewise do the pus-globules, and that very rapidly, with the aid of heat. The bile used by Dr. Hünefeld was that of the ox.

"If pus or puriform sputa be triturated together with bile, there results a gelatinous very viscous ropy fluid, not readily diffusible in water from its consistence, but which eventually, by being heated, dissolves into a slightly turbid liquor. When purulent serum is mixed with gall (freed from mucus by digestion in alcohol, &c.), filtered, then decomposed by alcohol and made to boil, a kind of loose coagulation and a muco-pulverulent deposit of albumen ensue. When, again, a solution of the pus-globules decomposed by alcohol is made to boil, no coagulation takes place, and even after long standing only a trifling sediment can be perceived. In this manner the mass of pus-globules can be completely distinguished from albumen and fibrin, for the solutions of these substances in bile will coagulate on the free addition of alcohol and the application of heat. As these corpuscules are the essential constituents of pus, the comportment towards bile forms a very important characteristic of pus, particularly in microscopico-chemical analysis. If mucus be taken by way of contrast (I employed the buccal mucus of man and the gastric mucus of pigs) it is reduced to a tenacious ropy liquid, and the vesicles disappear; if alcohol be added and heat employed, it then separates again exactly as happens when bile is so heated for removing its mucus, and the separated mucus rises in thick shreds to the surface of the liquor, presenting a grumous mass when examined with the microscope. This, therefore, affords a good criterion of pus and mucus." (p. 65.)

The circumstance that pus burns with a flame in consequence of the fatty matter it contains, supplies an excellent and ready test, first proposed by Güterbock.

The author adopts the view of Langlois, that tubercles consist essentially of fibrin.

"Tubercular substance heated along with concentrated hydrochloric acid forms presently a beautiful deep amethystine or lilac transparent solution, as is precisely the case with pure fibrin. Thus fibrin can be separated from albumen. It is prerequisite that the substance to be examined be freed from fat by means of ether, otherwise the solution will have a muddy brownish-red appearance." (p. 74.)

Under the head of chymification, or the alteration produced on food by the gastric juice, Dr. Hünefeld, after commenting upon the various opinions of physiologists, details a series of experiments instituted to prove the non-existence of free hydrochloric acid in that secretion. They are based upon the volatile nature of that acid. But we do not consider the negative evidence he has adduced sufficient to countervail the results obtained by so accurate an observer as Dr. Prout.

Wasmann's recent and elaborate researches upon pepsine, to which the author refers, place in a very strong light the solvent agency of hydrochloric acid. It appears to us that the true theory of digestion is that which attributes the *act of solution* to the *hydrochloric acid;* and the *preparation* of the aliment for that solution, to the pepsine or peculiar animal principle of the gastric fluid, which seems to act the part of a ferment, by causing such an incipient change in the substances to be digested as makes them more readily soluble in the dilute acid. This appears from the fact, that a long-continued moderate heat will enable hydrochloric acid to affect the solution without the pepsine; and that a small quantity of pepsine is sufficient to prepare for solution an almost unlimited amount of aliment, whilst the quantity actually dissolved depends upon the quantity of free muriatic acid in the liquor.

Our knowledge of the chemical constitution of the bile is at present very indeterminate. The experiments of Demarçay, and the conclusions to which he arrived, have been impugned by Berzelius. Our author having, as above stated, ascertained its energy in dissolving the blood-corpuscles, was led to try its action on various organic matters. The results are as follows:

"1. Bile reduces the fibrous clot of the blood to a clear liquor, in from a quarter to half an hour, at a temperature ranging between 95° and 100° Fahr. This is seen best betwixt two slips of glass, sealed together at the edges. 2. Bile dissolves casein (separated from milk by alcohol or a vegetable acid) under like circumstances, within the space of a few minutes; the cheesy substance obtained from sour milk is most rapidly dissolved. If that be triturated along with bile a thin pap is formed almost entirely soluble in water: the liquor is at first somewhat turbid, but becomes ere long transparent. The application of heat quickens the effect, but causes a creamy pellicle, consisting of butter, sometimes mixed with a little casein, to rise and float on the surface. The liquid itself is a combination of casein and bile, unaffected by heat, and from which acetic acid precipitates a mixture of casein and cholic acid. By the above property we can distinguish casein from coagulated albumen and from fibrin. The prompt solution of casein by bile, I hold to be an important fact. 3. Gall dissolves at the above temperature, in the course of a few hours, flesh grated and cooked; the experiment being conducted between slips of glass sealed together. The inflammatory crust of blood, under similar circumstances, was very soon reduced to a clear liquid." (p. 105.)

Ox-gall has been long reputed as stomachic; and if the above results be confirmed, its remedial agency may be ascribed in some degree to its favouring the solution of certain kinds of food in weakened states of the stomach and intestines. The kidneys stand in close relation to the liver, hence the bile may be considered as complementary to the urine. By the latter the blood is freed of saline matters, particularly ammoniacal salts and analogous combinations; by the former of fatty substances, cholesterine, margaric acid, choleic acid, and of biliary colouring matter, which last is probably the colouring matter of the blood partially modified. Haller says very justly, that the bile, if purely excrementitious, would pass, not into the duodenum, but into the rectum.

While treating of the changes produced upon food by the juices of the alimentary canal, Dr. Hünefeld points attention to the fact that the ammoniacal decomposition of mucus and its accumulation are frequently the cause of crystalline deposits in the intestines. Valentin found in the small intestines of a bear beautiful rhombic crystals of gypsum associated with organic matter. In the human subject, likewise, microscopic crystals have been detected, composed of phosphate of lime, sulphate of lime, and a salt of soda, namely, in the excrement of a typhous patient, a circumstance to which Schönlein has attached considerable weight. Gluge concludes, from more recent investigation, that crystals are very common in the fæces of typhous patients, and differ from those in the fæces of persons labouring under other diseases or in health. The excessive formation of ammonia may have something to do with this. Wittstein has shown that phosphate of lime is soluble in certain ammoniacal salts when aided by warmth.

The cecum contains both lactic and butyric acids. In this portion of the bowel there is carried on, to a certain extent, a repetition of what takes place in the stomach and small intestines; and its mucous membrane exhibits an acid reaction whenever the food contains much that is indigestible and stimulating. In the colon are found the insoluble remains of food, chiefly vegetable, brown colouring matter of the bile, mucus, fat, cholesterine, azotised extractive matter which acids redden, several soluble and insoluble salts (of soda, lime, and magnesia), besides fetid volatile products. (p. 120.)

In the chapter on respiration, we are told that the air remaining in the lungs after death consists for the most part of azote, with some carbonic acid. A solution of deoxidized indigo on being injected assumed merely a greenish tint: a new *docimastic* test is thus furnished. The residual air is retained with great force in the lung; the whole being expelled only after boiling in water, whereupon a lung which has breathed sinks to the bottom of the vessel. A large amount of air is thus obtained. The specific gravity of the lung which has breathed is about that of ether, therefore it floats in alcohol and oil of turpentine. The lungs can be washed with chlorine water, nay even steeped in it for some time, without losing their buoyancy; a thing of importance in a medico-legal inquiry.

The blood is fully considered. It is a singular fact, that various salts, namely, sulphate of soda, nitrate of ammonia, iodide of potassium, possess, under particular circumstances, the property of facilitating the mechanical separation of the coloured molecules from the *liquor sanguinis*, so that they can be easily removed by mere filtration. (p. 159.)

The saline ingredients of the blood are chiefly derived from the food. In the blood of the herbivorous animals there is a predominance of potash over soda salts, while in that of the annelides there appears to be very little soda. Sal ammoniac is stated by Lecanu to be present, but of this

Dr. Hünefeld is doubtful. The minute portion of ammoniacal salts which exist would seem to be only the lactate and phosphate. Berzelius, indeed, is of opinion that ammonia is seldom in the blood; and that where chemical analysis has revealed the existence of sal ammoniac, it is more than probable the result of the mutual decomposition of chloride of sodium and phosphate of ammonia. It is possible that more ammoniacal salt would be discovered in the blood were it not removed as soon as formed, as is the case with urea.

Ammonia is generated by the action of alkali upon protein and other animal matters, especially mucus; the alkali contained in the albuminate or fibrinate of the blood is sufficient to cause this.

"If serum, blood, serous fluids, and the like, be preserved from putrefaction with ether, and allowed to remain some time in a closely-corked glass phial, the liquid will be found to contain distinct traces of ammoniacal salts. Their proportion will be considerably augmented by a slight addition of caustic alkali. The best plan of proving the presence of ammonia is by triturating the liquor in question (when fresh) with hydrated oxide of lead or chalk, and then distilling along with alcohol. The product will strike a gray colour with protonitrate of mercury, provided there is any pre-existing ammoniacal salt. The constituents of the blood do not seem to modify the crystalline form of sal-ammoniac. I poisoned a rabbit with it (it swallowed daily a scruple, and lived sixteen days); the blood slowly dried between two slips of glass and examined with the microscope, presented distinct crystals of sal ammoniac. Through retention of ammoniacal salts in the blood, originate most probably violent symptoms of irritation, and thence a breaking up of the tissues; the alkali of the blood may also disengage some free ammonia." (p. 170.)

The potassium test lately suggested, would, we believe, set the ammoniacal question at rest.

Some practical directions are given for estimating the composition of the blood, followed by remarks concerning the leading peculiarities of arterial, venous, and portal blood, chiefly drawn from the observations of C. H. Schultz. The chapter winds up with an account of the changes effected on the blood by disease, as recorded by Lecanu, Mulder, and others, but without annexing anything novel or interesting.

In reference to what may be termed the analytical processes of the animal economy, namely, nutrition, secretion, and excretion, there prevails much diversity of opinion. Not a few writers entertain the notion, that all the substances from which the organic solids and fluids originate exist ready formed in the blood. The circumstance of urea being found in the blood after extirpation of the kidneys, and after mortification of the nerves going to the kidney as mentioned by Nysten, as likewise of its being discovered by Barrel and Marchuand in the fluid of dropsy, taken in conjunction with the fact of the prodigious velocity with which certain substances are thrown off from the blood, and the imperfections in the modes of chemical research, affords a fair ground for conjecture that various matters preexisting in the blood may have escaped notice. Agreeably to this view, the organs of secretion and excretion are merely channels of separation and not of formation, which is but the revival of the chemical pathology of a former age.

On the other side cogent arguments can be adduced to demonstrate that various matters have their being without the pale of proper *hæmatosis*, in the intimate texture of organs by the conversion of tissues, so to speak. To these appertain the primary constituents of bile, several modifications of fat, casein, sugar of milk, gelatine and chondrine, mucus, horn, elastic vascular texture, pigmentum nigrum and other colouring matters; in pathology, xanthic oxide, uric acid, cyanurine, &c. Of the above none have been as yet detected in the blood; though, perhaps it is true, seldom sought for with adequate skill and knowledge. (p. 189.)

On this we would remark, that of the existence of cholesterine and the colouring matter of the bile in blood there can be no doubt whatever; and that, in regard to others of the substances mentioned, there would be great difficulty in distinguishing them from the albuminous matter of the mass of the blood, on account of the absence of any definite characters. Some of the recent French analyses indicate horny matter and casein as substances to be more or less constantly detected in the blood.

The author, again reverting to the conversion of sugar into lactic acid and of vegetable salts into alkalis of the corresponding bases, thinks we may frame some hypothesis as to the power by which parenchymatous organs, especially those of the complex mucous glands, may act upon this or that constituent of the blood. Besides, the mechanically increased influence of alkali and oxygen on the one hand and of the generally acid fluid of parenchymatous organs on the other, must materially aid. All this reminds us a good deal of the reasoning and doctrines of Silvius de la Boe. He continues:

"The conversion of salts containing organic acids into carbonates is most likely the source of the alkali of the blood and various secretions; the albumen and fibrin evolving carbonic acid, albuminates and fibrinates result, which change is favoured by animal heat, capillarity, and other agents. The salt most uniformly subject to undergo conversion into carbonate is lactate of potash, and which is diffused throughout the whole organism." (p. 190.)

Several pages are occupied with the chemical qualities of the different textures and products of the body. Dropsical effusions are interesting in reference to pathology.

"1. These morbid collections possess in most instances the constituents of serum. 2. The albumen is identical with that of the serum, occasionally corresponding in quantity (7 to 8 per cent.) more seldom exceeding, most generally under (½—1 per cent.) 3. Hydropic fluid is now and then fibrinous, coagulating out of the body as observed by Magnus, in the inflammatory diseases of mucous membranes, and also thrown off along with false membranes, as in pleuritis. Tennant and Quevenne examined a fluid of this description from the pleura, the result of inflammation, which resembled concentrated lymph. 4. Not rarely, perhaps always, a substance analogous to the saliva, by some termed mucus, by others a peculiar uncoagulable substance, by others again of an earlier date, gelatine. It appears to be the source whence hydropic effusions poor in albumen appear thick and ropy, 5. According to some writers cholesterine has been found partly suspended in a fine crystalline form. Lassaigne and Marchand met with it in the fluid of hydrocele and also in that of sarcocele. 6. The dropsical fluid of an icteric patient contained the elements of bile and also cholesterine. Bouchardat sought in vain for the constituents of milk in that of a patient labouring under puerperal peritonitis." (p. 233.)

Buchner made the curious observation that during catarrh the secretion of the mucous membrane of the nose contains no nasal mucus, but is serous and saline; towards the termination of the affection it becomes again mucous, when it contains a peculiar acid volatile fat, also found in the expectoration of phthisical subjects. This may be at once recognized by its emitting flame when dried on a slip of platinum and exposed to the heat of a lamp.

It is well known that healthy urine when just voided gives an acid reaction. This is due partly to the presence of free lactic acid, partly to that of acidulous phosphates of ammonia and lime and uric acid. On account of the property which ammoniacal salts possess of reddening litmus when dried, urine is only fit for testing when fresh and unevaporated. It deserves notice that Berzelius has shown that butyric acid occasionally exists in urine. In the case of nurses and blond females it is uniformly present. This then is another source of acid reaction.

It was presumed that phosphoric acid taken into the stomach would render urine acid, when alkaline from disease. The illustrious chemist last named mentions a case where a calculous patient swallowed that acid in gradually increased doses. The urine was at first not acidulous, but eventually the phosphoric acid caused diarrhea, whereupon the urine became acidulous and clear (which it was not previously) and deposited uric acid. This ceased with the diarrhea, and neither the continued use of phosphoric acid nor of acetic acid counteracted the sediment or the alkaline condition of the urine; the patient progressively wasted away and died. (p. 273.) The phosphoric acid therefore proved a failure. From Mr. Ure's recent researches it would appear that benzoic acid renders alkaline urine acidulous and limpid in virtue of the formation of hippuric acid, and thereby obviates calcareous deposition. (Med. Gaz. February, 1843.) This certainly is an important step as regards the pathology of the urine.

Various modifications in the nature of the urine produced by diseased action are pointed out, but without either order or novelty. Under the directions for testing the urine we find the following recommended for ascertaining the degree of acidity: to a given measure of the fresh urine, tincture of litmus previously brought to a state of perfect neutrality by means of acetic acid is to be added, and then liquid ammonia of a definite strength to be slowly poured into the mixture from a graduated glass vessel until it assumes a blue tint; from the quantity of ammonia expended the precise amount of acid may be deduced. Or a certain weight of ignited carbonate of soda dissolved in water may be used. This is to be gradually dropped into the urine (slightly warmed) until it becomes quite indifferent to test (red and blue litmus) paper. Tincture of turmeric darkened with ammonia can also serve as a means of acidimetry, but the manipulation requires some practical experience. (p. 245.)

Numerous processes have been at different times proposed for determining the proportion of sugar in diabetic urine. Perhaps the most accurate is that of Simon, which Hünefeld has not supplied. It directs the urine, previously evaporated to the consistence of syrup, to be mixed with from four to six times its weight of anhydrous alcohol; this precipitates the greatest part of the sugar, which is next to be washed with a fresh quantity of alcohol. It is now to be dissolved in a little water, and alcohol added in order to precipitate the mucus, salts, &c. The solution is to be filtered, evaporated to dryness, and the sugar dissolved in strong boiling alcohol, from which it is deposited by slow evaporation in mammillary groups of minute crystals. The same author recommends Von Trommer's method for finding out sugar in the blood. The protein combinations are to be precipitated with anhydrous alcohol; to the alcoholic solution some dry carbonate of potash is to be put and the mixture well shaken. A small quantity of solution of sulphate of copper is then to be added and heat applied. If sugar be present, a yellow or yellowish-brown film of protoxide of copper will form in the undermost layer where the potash is diffused.

While on the subject of the urine, M. Lecanu's mode of detecting minute portions of blood deserves notice, as it is a thing of considerable importance and difficulty. He directs us to bring the urine to the boiling point so as to coagulate the principles of the blood, which are afterwards to be collected on a filter, washed, and then squeezed. They are next to be boiled in alcohol containing a drop of sulphuric acid. This dissolves out the hematine, which may be separated by alcohol in the usual way, and at once recognized, after calcination, by its red ferruginous ash. The hematine separated under these circumstances floats upon the surface of the liquor as a black resinous mass, which forms a red solution with acetic acid and ammoniated alcohol.

The third section of this book treats of the chemical comportment of the inorganic and organic matters to the organic fluids, and of these to each other. We subjoin his observations regarding the ammoniacal salts.

"The officinal and probably all the salts of ammonia have the property to a greater or less degree of dissolving the blood-corpuscles even to the nucleus, although slowly, and the protein textures generally. Whether they are thus endowed of themselves or whether it is in virtue of ammonia set free by the alkali of the blood, is the question; at all events we have already ascertained that free ammonia is not essential to these effects. When the blood is combined with an ammoniacal salt it acquires generally a brighter red, but this soon passes into a brownish-red hue; it does not coagulate, or forms at best a mere loose semi-fluid cruor, the corpuscles begin to disappear and the whole becomes more limpid. Succinate of ammonia, however, seems to form an exception, for by the admixture of one part in a hundred the clot remained firm, friable to the feel, and of a nut-brown aspect. Of all the salts tried the cyanate of ammonia (which by evaporation yields urea) exercised the most powerful solvent agency. Hydrochlorate of ammonia renders the blood clearer and heightens its colour more than the other salts; under its influence the blood-corpuscles disappear very quickly. Nitrate of ammonia in excess imparts to the blood a beautiful vermilion-red tint and precipitates the corpuscles rapidly and completely before they are dissolved, so as to allow the clear serum to be readily filtered, ere long solution commences and is fully accomplished at the ordinary temperature. Blood thus decomposed progressively evolves distinct traces of ammonia. It is very probable that we may partially explain upon chemical grounds (solution and disengagement of ammonia) why large doses of sal-ammoniac act as poisons, and smaller doses long continued induce a scorbutic condition, yet the same medicament judiciously exhibited furnishes a valuable stimulant to the secretory and excretory apparatus. That chemical attraction is inadequate to account for the therapeutic and toxico-dynamic power of sal ammoniac is obvious, inasmuch as it exercises a general action and induces inflammation of the stomach, even when introduced into the subcutaneous cellular texture. Frogs, owing to the extreme delicacy of their intestinal mucous membrane, die usually with excessive rapidity after trifling doses of this salt: placed on the dead gut it renders its surface slippery and the mucous coat swollen." (p. 281.)

Dr. Hünefeld observes that urea (anomalous cyanate of ammonia) likewise dissolves the blood-corpuscles.

Grognier discovered sal ammoniac in the serosity of a horse which had been poisoned by its means. Whether ammoniacal salts really undergo decomposition in the blood, ammonia being set free, or whether they are carried off by some of the emunctories, as the kidneys or the skin, requires further investigation. They no doubt, like other salts, possess the property of dissolving the albuminous elements of the blood, and thus destroying their plastic nature. Hence their utility in inflammatory diseases where the amount of fibrin is notably augmented.

Medical men have generally looked to chemistry to supply them with antidotes against poisons, and they have not looked in vain. Libavius long ago remarked with great truth, " Est quidem chemia in hoc famula medicinæ ad modum solers, sed tantum non sola illud obtinet, et requirit prudentem artificem, qui possit salvo auxilio nocitura tantum tollere, sciatque qua in parte sit utrumque " (Tr. de veneno sing. P. i. 1599). The discovery of hydrated oxide of iron as an antidote for arsenic, by MM. Berthold and Bunsen, and of the mixture of iron-filings and gold-dust as a *galvanic* antidote for corrosive sublimate by Dr. Buckler, are unquestionably important achievements in the department of toxicology. In reference to the former, Duflos recommends what is termed the *liq. ferri acet. oxyd.* as being preferable to the hydrated oxide, inasmuch as that is only available in cases of poisoning from arsenious or arsenic acid, but not so where arsenite of potash has been swallowed, such as exists in Fowler's solution. Hence he advises, whenever any doubt is entertained concerning the precise nature of the arsenical preparation, that the acetate be administered so as to give the patient the benefit of that doubt. It is prepared with one part of hydrated oxide of iron, three parts of vinegar of specific gravity 1·06, and as much water as will make in all sixteen parts. An excess of acetic acid is no ways injurious.

The fourth section is prolix and confused. It is headed, " physiological bases of pharmacology, as an interponent between organic chemistry and special therapeutics." It comprehends articles of diet, water, atmospheric air, warmth, electricity, and disease. The fifth and last section is entitled, " a criticism of the materia medica, in reference to its chemical and physical principles." A new classification is introduced, including: 1. Neutral assimilative substances. 2. Mechanical agents. 3. Excitants. 4. Solvents. 5. Decomponents. The author conceives that the metallic salts and oxides exercise a direct decomposing influence upon the blood and other circulating fluids, but he is unable to assign any sufficient chemical reason for their remarkable toxico-dynamic and pharmaco-dynamic effects. He is, on the contrary, more inclined to ascribe the different changes produced to nervous influence.

> " Iron alone is a uniform constituent of the organization, all other metallic combinations invariably act in a deleterious manner upon the animal organism; their very taste shows how heterogene they are. They ere long induce a state of decomposition incompatible with life, the primary cause of which seems to be nervous irritation. It would appear that the ganglionic system is the one to which the dynamic effects of metallic preparations are immediately directed. Hence many cases of deadly poisoning have been known to occur from their administration, in which there was no sign of inflammation." (p. 344.)

He very properly censures those persons who imagine that chemistry can and must lead to an *à priori* knowledge of the action of remedies, and then run it down as futile because it fails to do so. They overlook what life is, what organization is, what chemistry is. They do not think that if chemistry could accomplish this, it would be essentially medicine. Each external substance, he observes, can act upon the organism mechanically, dynamically, and chemically ; but it is only the dynamic agency with which the medical man has to do, independently of surgery and toxicology. C. G. Vogel emphatically declared " half the materia medica is uncertain and full of contradictions," and thus it will remain unless made the subject of rigorous inductive inquiry. Professor Marx says, " were every medical man, or a union of medical men, to select for trial and close investigation during a series of years a single remedy only, and were their results reciprocally communicated and afterwards tested in other places under other conditions, we should then have a materia medica upon which reliance could be placed, when each tried noxious medicament could be exhibited without risk of peril. Until this work is accomplished, the conscientious practitioner can resort to but few remedies; for whenever the choice lies between what is harmless and what heroic, he must unconditionally employ the former."

Taken as a whole the work before us is certainly a most heterogeneous compound, containing here and there useful information, but extremely deficient in clear and consecutive reasoning. The author is evidently a very laborious compiler, for there are nearly three hundred references put down at the end of each " book." Yet the facts are so ill assorted, and there is so much loose hypothetical assumption, as to convey to the reader's mind an impression far from satisfactory. Indeed, it requires no ordinary share of patience to get through these volumes.

